# Dose–effect of long-snake-like moxibustion for chronic fatigue syndrome: a randomized controlled trial

**DOI:** 10.1186/s12967-023-04250-z

**Published:** 2023-07-03

**Authors:** Hong Luo, Rui Gong, Rui Zheng, Jing Tan, Ruixue Chen, Jie Wu, Tingting Ma

**Affiliations:** 1 College of Acupuncture and Tuina, Chengdu University of Traditional Chinese Medicine, Chengdu, 610075 China; 2 Center of Chinese Evidence-Based Medicine, Sichuan University West China Hospital, Chengdu, 610041 China; 3 Center of Preventive Medicine, Hospital of Chengdu University of Traditional Chinese Medicine, Chengdu, 610075 China

**Keywords:** Chronic fatigue syndrome, Long-snake-like moxibustion, Dose–effect relationship, Randomized controlled trial

## Abstract

**Background:**

The dose–effect relationship of Long-snake-like moxibustion for chronic fatigue syndrome (CFS) remains poorly understood. In order to address this gap, we designed this trial to assess the association between different treatment duration of Long-snake-like moxibustion and its effects on CFS based on the combination measurements of the subjective patient-reported scales with objective medical infrared imaging technology─Thermal Texture Maps (TTM).

**Methods:**

From December 2020 to January 2022, 60 female CFS patients were recruited and equally allocated to two groups: Group A, receiving 60-min Long-snake-like moxibustion per treatment, and Group B, receiving 30-min Long-snake-like moxibustion per treatment. The treatment was administered 3 times per week for a total of 4 weeks. The primary outcome was defined as the improvement of symptoms measured by the Fatigue scale-14 (FS-14), and secondary outcomes were designated as the improvement in Symptoms Scale of Spleen-Kidney Yang Deficiency, Self-rating depression scale, and Self-rating anxiety scale. TTM scanning was employed twice for CFS patients (before and after 4-week treatment) and once for Healthy control subjects (HCs).

**Results:**

At week 4, the scores of FS-14 and Symptoms Scale of Spleen-Kidney Yang Deficiency in Group A were significantly lower than those in Group B (physical fatigue: 5.00 vs. 6.00, *with* 95%CI − 2.00 to 0.00, *p* = 0.003; FS-14 total score: 8.00 vs. 9.00, *with *95%CI − 3.00 to 0.00, *p* = 0.012; total score of Symptoms Scale of Spleen-Kidney Yang Deficiency: 9.80 vs. 13.07, *with *95%CI − 5.78 to − 0.76, *P* = 0.012). All thermal radiation values of the two groups increased, and statistical differences in ΔTs between Group A and HCs were not obtained. More significant correlations between symptoms improvements and ΔT changes were observed in Group A, and its ΔT changes in Upper Jiao, Shenque (CV8), Zhongwan (CV12), Danzhong (CV17), Zhiyang (GV9), Dazhui (GV14), upper arm, thoracic segments, lumbar segments, renal region, popliteal fossa strongly correlated with the improvement of Spleen-Kidney Yang Deficiency symptoms.

**Conclusions:**

In the same course of treatment, the positive dose–effect relationship was found between the treatment duration of Long-snake-like moxibustion and CFS effect assessment. 60-min Long-snake-like moxibustion per treatment were associated with optimal clinical response and TTM improvement.

***Trial registration*:**

Chinese Clinical Trail Registry (No. ChiCTR2000041000, date of registration: 16 December 2020), http://www.chictr.org.cn/showproj.aspx?proj=62488

**Supplementary Information:**

The online version contains supplementary material available at 10.1186/s12967-023-04250-z.

## Background

Chronic fatigue syndrome (CFS) is a condition characterized by persistent and debilitating fatigue lasting for at least 6 months, which is not relieved by rest [1]. The fatigue and its accompanied symptoms, including sleep disturbance, pain, sore throat, impaired short-term memory and concentration, greatly affect patients’ quality of life and ability to work. CFS has a global prevalence of 40% and is more prevalent in women than in men [2]. Despite its widespread occurrence, the medical cause of CFS is still unclear, resulting in a lack of specific pharmacological treatment recommendations. However, several promising approaches, including acupuncture [3], moxibustion [4], yoga [5], cognitive behavioral therapy), and graded exercise therapy [6–8] have shown potential for treating CFS.

According to the theory of traditional Chinese Medicine (TCM), fatigue is the result of Yang deficiency. Yang, or Yang-qi, an important concept in TCM, represents the vital energy of life. Yang deficiency refers to a pathological state characterized by a deficiency of Yang qi, representing the impaired functions of warming and promotion. Consequently, when organ function declines, the symptoms such as decreased vitality, listlessness, aversion to cold and preference for warmth, and reduced metabolism will manifest. The most typical affected Zang and Fu of Yang deficiency is Spleen-Kidney Yang. Moxibustion has the ability to produce Yang-warming effect and improve deficient Yang for Zang and Fu. Although the precise mechanism by which moxibustion treats CFS is not fully explained, moxibustion therapy has been shown to be effective in relieving symptoms [9, 10]. Long-snake-like moxibustion, also known as Du moxibustion, involves applying a large range of moxa cones or sticks over herbs or ginger on the Governor Vessel from GV14 to GV2 to generate a powerful warming stimulation. Compared to general moxibustion therapies, Long-snake-like moxibustion has a wider treatment area and utilizes the medicinal properties of moxa wool and ginger. It has a greater effect of warming yang, supplementing qi, promoting the circulation of qi and blood, and harmonizing yin and yang. Currently, there are a variety of Long-snake-like moxibustion applications. It has been applied to rheumatic immune diseases [11], osteoarthropathy [12], digestive disorders [13], etc. The mechanisms behind the therapeutic effects of Long-snake-like moxibustion on these diseases may involve the regulation of immune imbalance, reduction of immune response, acceleration of blood circulation, alleviation of tissue edema, and reduction of gastrointestinal burden. In our previous study [14], we improved the Long-snake-like moxibustion technique through applying moxa boxes placed over ginger slices on the back from GV14 to GV2, as well as on the same level of the first lateral line of Bladder Meridian. This modification enhanced clinical operability and improved the therapeutic effect.

We searched several databases (PubMed, Embase, Web of Science, Chinese National Knowledge Infrastructure Database, Wanfang Database, China Science and Technology Journal Database, etc.) from their respective inception dates to January 31, 2020. The search was performed using keywords such as ‘moxibustion’, ‘moxibustion therapy’, ‘moxibustion treatment’, ‘dose’, ‘moxibustion dose’, ‘dose–effect relationship’, ‘moxibustion effect’, etc. The searching results found that moxibustion is influenced by various factors that affect its therapeutic efficacy, such as moxibustion dose, which includes moxibustion duration, treatment frequency, moxibustion amount-size, and the number of moxa cones used [15–18]. Therefore, we conducted this trial to investigate the relationship between the duration of Long-snake-like moxibustion and its effect on CFS by using both subjective scales and objective measurements for assessment.

So far, CFS trials evaluating the efficacy and safety of therapies have employed self-reported subjective measurements, including the Chalder Fatigue Scale, Short Form 36 Health Survey Questionnaire, Anxiety and Depression Scale, and Visual Analogue Pain Rating Scale, to evaluate improvements in physical function, anxiety, depression, and pain [19]. In addition, biological markers and imaging techniques, such as MRI, have also been used to support clinical diagnosis and evaluate treatment effects [20, 21]. In this trial, we used Thermal Texture Maps (TTM), a thermography technique that evaluates thermal signatures of the body through a holistic interpretation of infrared images, to aid in CFS diagnosis and moxibustion dose–effect assessment.

## Methods

### Design

The single-center, randomized, controlled trial was conducted at the Center of Preventive Medicine, Hospital of Chengdu University of TCM. This trial strictly adhered to the rules of the Declaration of Helsinki and the Good Clinical Practice Guidelines, and it was registered with a unique registration number (ChiCTR2000041000) at http://www.chictr.org.cn. The protocol received approval from the Ethics Committee of Affiliated Hospital of Chengdu University of TCM in 2020 (No.2020KL-046). Written informed consent was obtained from all participants, which included 60 female patients with CFS and 30 healthy control subjects (HCs).

The CFS patients were randomly allocated to either the 60-min Long-snake-like moxibustion group (Group A) or the 30-min Long-snake-like moxibustion group (Group B) in a 1:1 ratio, with a treatment period of 4 weeks consisting of 3 times per week, every other day.

### Subjects

Patients were recruited from multiple sources, including the hospital as well as electronic posters on WeChat and physical posters displayed in hospitals and communities. Face-to-face interviews were conducted by the clinical trial coordinator to ensure that all patients met the inclusion criteria. The inclusion and exclusion criteria for patients and HCs are shown in Table 1.

**Table 1:** The inclusion and exclusion criteria for patients and HCs

Patients with CFS
Inclusion criteria
1. Females aged 18 to 60 years old;
2. Chronic fatigue is lasting and can’t be explained by illness;
3. Debilitating fatigue predates and is accompanied by at least 4 out of the 8 designated symptoms: post-exertional malaise lasting more than 24 h; unrefreshing sleep; impaired short-term memory or concentration severe enough to cause substantial reduction in previous levels of occupational, educational, social, or personal activities; headaches of a new type, pattern, or severity; muscle pain; multi-joint pain without swelling or redness; sore throat; and tender cervical/axillary lymph nodes. Accompanying symptoms must have persisted or recurred during 6 or more consecutive months of illness.
Patients should also meet the inclusion criteria of the pattern of Spleen-Kidney Yang deficiency, which include
1. Presence of at least 3 out of the 5 main symptoms: fear of cold and cold extremities, lassitude and lack of strength, shortness of breath and laziness to speak, reduced food intake, soreness and weakness of waist and knees;
2. Presence of at least 2 out of the 6 accompanied symptoms: lumbar cold pain, abdominal fullness and distention, loose stool, cold, more urine volume during night, teeth-mark tongue and a deep, weak pulse.
Exclusion criteria
1. Pregnancy or lactation;
2. Presence of diseases that definitely influence TTM results, such as scoliosis or limb deformity, etc.;
3. Poor understanding or expression;
4. Presence of obviously damaged, scarred skin, or sensitivity to ginger.
HCs
Inclusion criteria:
1. Females aged 18 to 60 years old;
2. No disorder in physical examination in the preceding one year, and in good physical and mental health during the study period;
3. No family history of mental or nervous system disorders;
4. No spinal diseases that definitely influence TTM results, such as scoliosis, limb deformity, etc.
Exclusion criteria
1. Pregnant or lactating;
2. Presence of obvious damage or scarring on the skin;
3. Long term smoking, alcoholism, or adverse drug use;
4. Participation in another concurrent research study.

### Randomization and blinding

Eligible CFS patients were randomly allocated to either Group A or Group B. Random numbers were generated by Statistics Analysis System, and then placed in opaque envelopes. A research assistant, who was uninvolved in the assessment or treatment, was responsible for keeping the original random allocation sequences, printing serial numbers on the outside of the envelopes, and sealing the envelopes. All envelopes were put into a plastic container in numerical order. Once patients were assessed to meet the inclusion criteria and provided informed consent, investigators opened the envelopes in turn. To ensure blinding, the outcome assessors and statistical analysts were blinded to the intervention assignments throughout the trial.

### Intervention

Practitioners: Certified acupuncturists with TCM license and more than 2-year clinical experience were trained to administer Long-snake-like moxibustion. The training focused on the correct manipulation of the moxibustion technique.

**Fig. 1:**
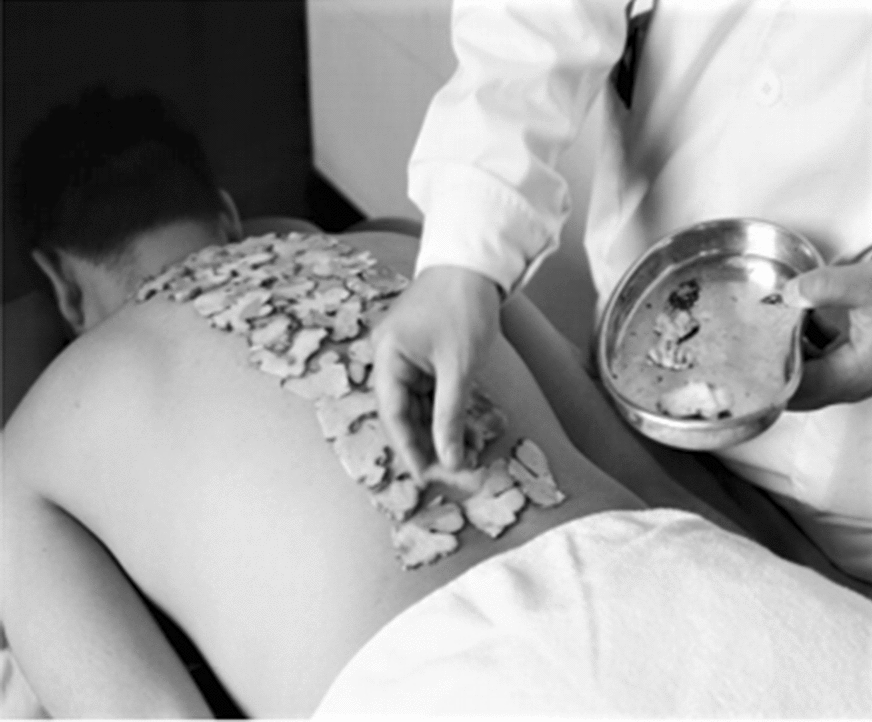
Ginger slices were placed on the back from GV14 to GV2

**Fig. 2:**
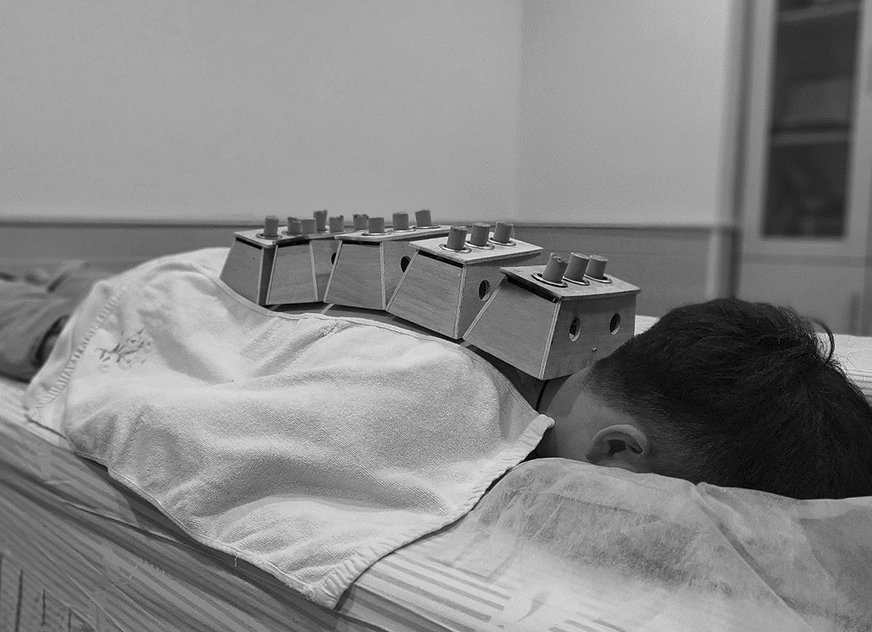
Five or six 3-hole moxa boxes were put on the participants of two groups

Group A: 60-min Long-snake-like moxibustion. Patients were positioned prone with their entire back exposed and received ginger-indirect moxibustion on the Governor Vessel and Bladder Meridian. Ginger slices, 2 mm in thickness, were placed on the back from GV14 to GV2, covering the area of the Governor Vessel and the first lateral line of the Bladder Meridian (Fig. 1). Five or six 3-hole moxa boxes (depending on the patient’s height, Fig. 2) were then placed on top of the ginger slices, with a distance of 4.8 cm between the centers of the nearby holes to ensure the moxa sticks’ temperature (pure-mugwort moxa stick; 20 cm in length and 18 mm in diameter; Hwato, Suzhou, China, PRC) covered the Governor Vessel and the first lateral line of the Bladder Meridian. The depth of moxa sticks was regulated every 5 min during 60-min treatment to maintain full combustion and obtain equal warm stimulation. Patients received 12 sessions of moxibustion treatment over a 4-week period (1 session per day, 3 sessions per week, every other day).

Group B: 30-min Long-snake-like moxibustion. The treatment process was the same as in Group A, with a moxibustion duration of 30 min.

### Outcome measures

Primary outcome: In this trial, the improvement in fatigue severity assessed by Fatigue scale-14 (FS-14) was set as the primary outcome. FS-14 is a standardized questionnaire reflecting physical and mental fatigue and comprises 14 questions. Each question has two options (yes or no) and a score of 0 to 1 (0 = no, 1 = yes), resulting in a total score ranging from 0 to 14 [22, 23]. Higher scores indicate a higher level of chronic fatigue. The assessment using FS-14 was conducted at baseline and at the end of the 4-week treatment period (week 4).

Secondary outcome: The secondary outcome measures included the scores of the Symptoms Scale of Spleen-Kidney Yang Deficiency, the Self-rating depression scale (SDS), and the Self-rating anxiety scale (SAS). The Symptoms Scale of Spleen-Kidney Yang Deficiency is a 4-rate scale designed to evaluate symptoms based on TCM syndrome differentiation. It consists of primary symptoms with scores ranging from 0 to 6, including fear of cold and cold extremities, lassitude and lack of strength, shortness of breath and laziness to speak, reduced food intake, soreness and weakness of waist and knees. It also includes secondary symptoms with scores ranging from 0 to 3, including lumbar cold pain, abdominal fullness and distention, loose stool, cold and more urine volume during night. Higher scores indicate a higher level of Spleen-Kidney Yang Deficiency. Both the SDS and SAS are 20-item scales designed to measure depression/anxiety with scores ranging from 20 to 80. Higher scores indicate a higher level of depression and anxiety. All secondary outcomes were assessed at baseline and at the end of the 4-week treatment period (week 4).

TTM scanning: 90 eligible participants (30 female CFS patients in Group A, 30 female CFS patients in Group B, and 30 HCs) received TTM (Digital Medical Infrared Imaging System, MTI-ex pro-2013, Chongqing, China) scanning at baseline, and only patients in Groups A and B received a second TTM scanning after the 4-week treatment period. To weaken the influence of external thermo-source on the moxibustion effect assessment, the second TTM scanning was employed 5 days after the end of treatment. To minimize the impact of outside temperature, all the scanning was executed in the morning at the health examination center of the Affiliated Hospital of Chengdu university of TCM. The scanning room was maintained at a controlled temperature of 21℃ ± 2℃ and a humidity level of 50% ± 10%. The subjects were positioned in an environment with no direct ventilation, sunlight, or infrared radiation source [24, 25]. The TTM scanning process and relevant precautions are detailed in Table 2, and the image area definitions based on the Three-Jiao theory in TCM (Upper Jiao, Middle Jiao, and Lower Jiao) are shown in Table 3 [26, 27].

**Table 2:** The process and relevant precautions of TTM

The process was listed below:
1. Fill the personal health information form before scanning, which contained the medical history and the state of body condition in all organs;
2. Remove the ornaments, loosen the bra, and relax hair in advance. Have a rest of 30 min at rest room before scanning;
3. Prior to the scanning, patients were asked to take all clothing off and expose the body in a temperature-controlled room for 10–15 min. Then images were taken from the front vs. back, and left vs. right to assess overall topological features of the whole body.
All participants were asked to adhere to the following precautions
1. Fasting for at least 2 h before the scanning;
2. Before the examination, alcohol should be stopped for at least 24 h, the usage of medications (i.e., vasodilator, vasoconstrictors) should be stopped for at least 4 h, vigorous exercise should be forbidden for at least 4 h, other stimulants should be stopped for at least 2 h and hand washing should be avoided for at least 30 min;
3. Skin conditioning such as use of cosmetics and perfume had to be avoided, and a dress of looser clothing is advised;
4. No scanning during menstruation.

**Table 3:** The definition of Three-Jiao in TTM image

Areas	Border
Upper Jiao	Upper border: the line of clavicles
	Lower border: the line of the 5th intercostal spaces
	Two sides: the anterior axillary lines
Middle Jiao	Upper border: the line of the 5th intercostal spaces
	Lower border: level with the umbilicus
	Two sides: the anterior axillary lines
Lower Jiao	Upper border: level with the umbilicus
	Lower border: level with the pubic symphysis

### Sample size

Based on our preliminary study [28], which demonstrated an improvement of 2.45 in the FS-14 score following 60 min of Long-snake-like moxibustion therapy, we expected a mean improvement difference of 0.4 in the FS-14 score. With a desired power of 0.9 and a significance of 0.05, the estimated sample size required was 27 participants per group. Considering a dropout rate of 10%, the total number of patients was 60, while the total number of HCs was 30.

### Statistical methods

The data was analyzed by an independent statistician. Quantitative data were described by mean with standard deviation (SD) or median with percentile (Q1, Q3). All the statistical analyses were performed with SPSS software version 25.0 (SPSS Inc, IBM Corporation, Chicago, Illinois, USA). The correlation analysis and visualization were performed with R language version 4.2.0 (R Development Core Team, Vienna, Austria). A two-sided test was applied for all available data, and a *P-value* < 0.05 was considered statistically significant.

The main analysis was based on an intention-to-treat population, and missing data at random were replaced by the multiple imputation method. The primary outcome of FS-14 score was analyzed by Mann-Whitney U-test. For secondary outcomes, including the score of Symptoms Scale of Spleen-Kidney Yang Deficiency, SDS score and SAS score, we conducted independent sample T test or Mann-Whitney U test (used in non-normally distributed variables) to compare the scores after 4 -week treatment between Groups A and B.

TTM analysis: Disease-related areas, specific regions or acupoints were acquired through comparing HCs with CFS patients in the whole-body thermal map. The relative thermal radiation value difference was defined as the temperature difference between the local temperature and the average temperature of the front or back of the trunk. It was automatically detected by TTM. Median differences and 95% confidence intervals of the temperature difference (ΔT) comparison between Group A or B and HCs at the end of 4-week treatment in the above area or acupoints were calculated by Hodges-Lehmann method.

Attrition analyses: Considering the possible effect from visit lost on results, we added 10% more sample size for each group in recruitment to hedge against expected attrition. For incomplete outcome data, we used multiple imputation method to replace random missing data. Sensitivity analysis using the per protocol set was performed and compared with the intention-to-treat analysis results.

### Data collection and quality control

The original data were recorded in paper case report form (CRF) and then filled in a spreadsheet. An independent investigator checked the original data to ensure the consistency of CRF and spreadsheet. Only the principal investigators and clinical investigators were qualified to review the data. Before the start of trial, all involved researchers underwent training to guarantee the full understanding of study protocol, research process and CRF-filling. Additionally, once the eligible participants were enrolled, full communication about possible benefits and risks were made and a comfortable treatment environment was provided to ensure participants’ compliance. For participants with poor compliance, investigators contacted them by phone to acquire the outcome data, inquire about possible reasons for non-compliance, and encourage them to complete the study.

## Results

### Patient characteristics

**Fig. 3:**
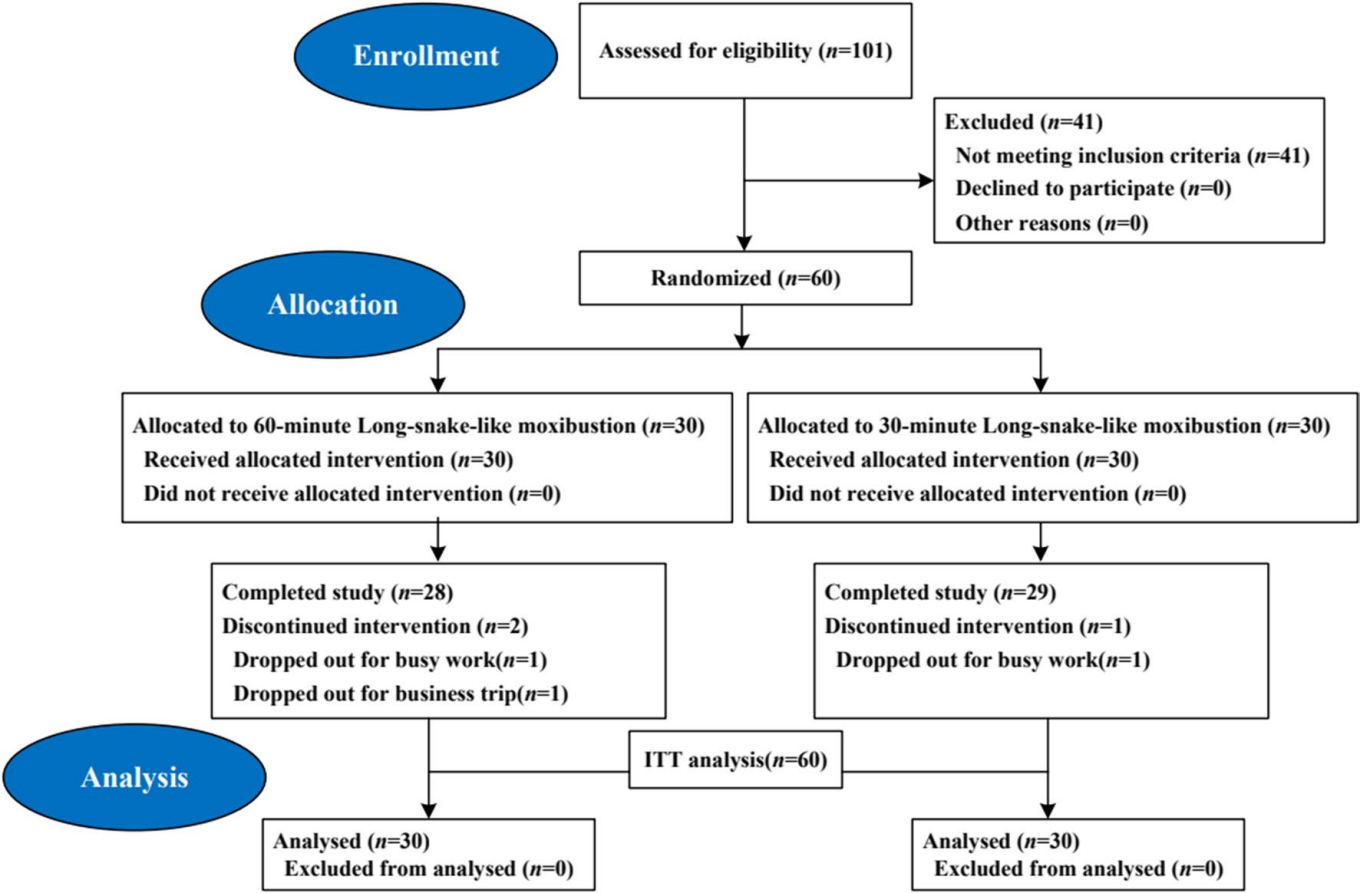
Patient flowchart. *ITT* intention-to-treat

From December 2020 to January 2022, a total of 101 patients were screened, and 60 patients were included with a median age of 30.50 (Q1, Q3: 24.00, 40.75) years and a median CFS duration of 31.00 (Q1, Q3: 12.00, 60.00) months. The study flowchart is shown in Fig. 3. There were no significant differences between the two groups with respect to baseline demographic and clinical characteristics (*P* > 0.05). These results suggested that the two groups were comparable (Table 4).

**Table 4:** Baseline characteristics of patients

Characteristics	Group A (n = 30)	Group B (n = 30)
Age (years), Median (Q1, Q3)	31.50 (24.00, 47.50)	29.50 (24.00, 38.50)
Course of disease (month), Median (Q1, Q3)	36.00 (24.00, 72.00)	24.00 (12.00, 48.00)
BMI (kg/m^2^), Median (Q1, Q3)	21.16 (19.64, 22.52)	21.12 (19.30, 22.85)
FS-14 at baseline, Median (Q1, Q3)		
Physical fatigue	7.50 (6.00, 8.00)	7.00 (7.00, 8.00)
Mental fatigue	4.00 (3.00, 5.00)	4.00 (3.00, 5.00)
Total score	11.00 (9.00, 12.00)	11.00 (10.00, 13.00)
Symptoms scale of spleen-kidney Yang deficiency at baseline, Median (Q1, Q3)		
Fear of cold and cold extremities	4.00 (4.00, 6.00)	4.00 (4.00, 4.00)
Lassitude and lack of strength	3.00 (2.00, 4.00)	2.00 (2.00, 4.00)
Shortness of breath and laziness to speak	2.00 (2.00, 4.00)	2.00 (2.00,4.00)
Reduced food intake	2.00 (0.00, 4.00)	2.00 (0.00, 2.00)
Soreness and weakness of waist and knees	2.00 (0.00, 4.00)	2.00 (0.00, 3.00)
Lumbar cold pain	1.00 (1.00, 2.00)	1.00 (0.00, 2.00)
Abdominal fullness and distention	1.00 (1.00, 2.00)	1.00 (1.00, 2.00)
Loose stool	1.00 (0.00, 1.00)	1.00 (0.00,2.00)
Cold and more urine volume during night	0.00 (0.00, 1.00)	0.00 (0.00, 1.00)
Total score, Mean (SD)	18.03 (6.39)	16.57 (3.86)
SDS at baseline, Mean (SD)	52.77 (11.76)	49.83 (10.22)
SAS at baseline, Mean (SD)	47.37 (11.83)	43.67 (9.09)

Group A: Long-snake-like moxibustion for 60 min; Group B: Long-snake-like moxibustion for 30 min; Data were presented as mean ± standard deviation (SD) and Median (Q1, Q3); Q: quarter; Q1: 25th percentile; Q3: 75th percentile

*BMI* Body Mass Index; *FS-14* Fatigue Scale-14; *SDS* Self-rating Depression Scale; *SAS* Self-Rating Anxiety Scale

### Primary outcome

**Fig. 4:**
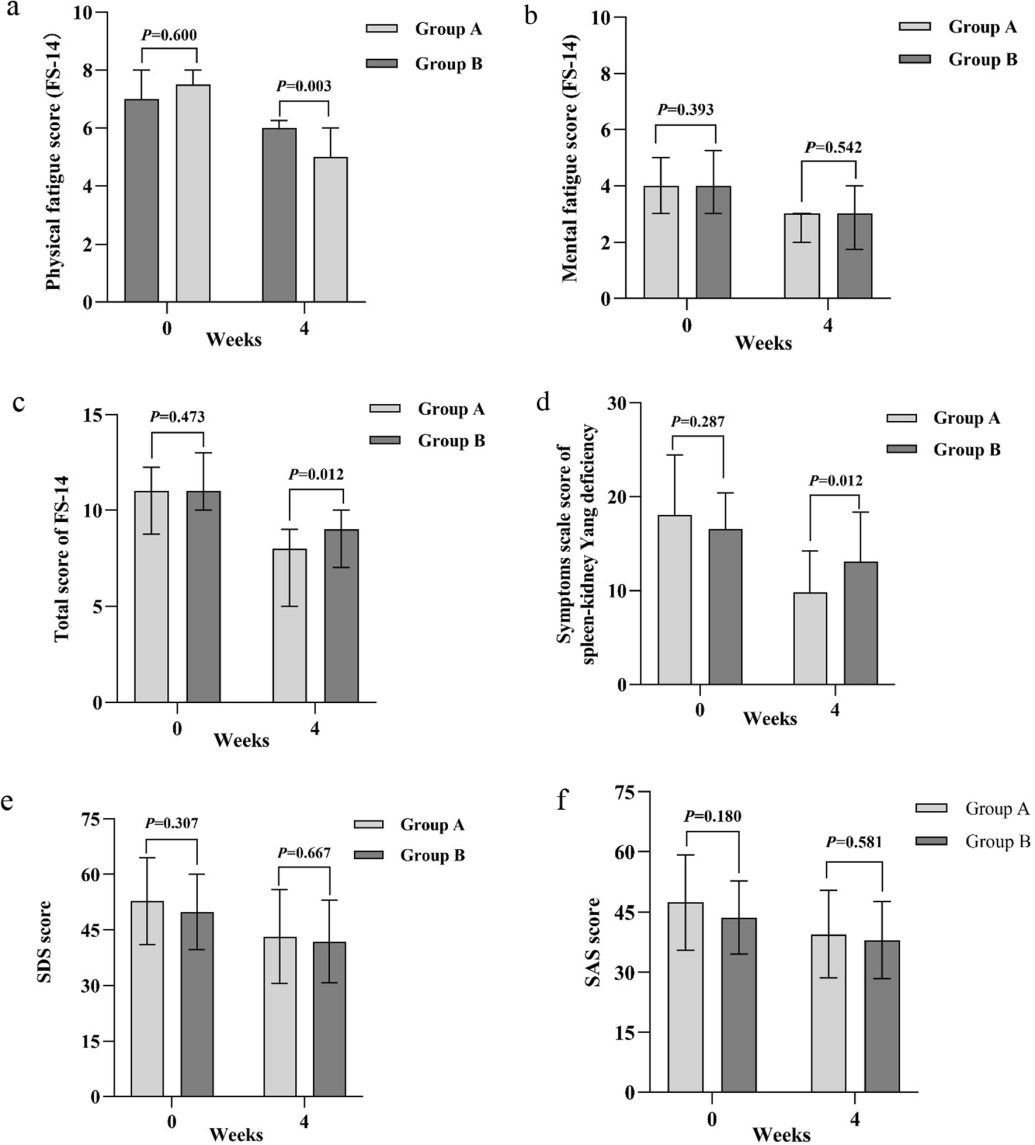
Effects of Long-snake-like moxibustion at baseline and week 4. Data in a, b, c were shown by the median with interquartile range and data in d, e, f were shown by mean with error bar represented standard deviation; **a** Physical fatigue score at baseline and week 4; **b** Mental fatigue score at baseline and week 4; **c** Total score of FS-14 at baseline and week 4; **d** Symptoms scale score of spleen-kidney Yang deficiency at baseline and week 4; **e** SDS score at baseline and week 4; **f** SAS score at baseline and week 4

After 12 treatment sessions within 4 weeks, physical fatigue score and FS-14 total score decreased in both groups compared to baseline (Fig. 4). Group A showed significantly lower scores at the end of treatment (physical fatigue: 5.00 vs. 6.00, 95%CI − 2.00 to 0.00, *p* = 0.003; FS-14 total score: 8.00 vs. 9.00, 95%CI − 3.00 to 0.00, *p* = 0.012) (Table 5).

**Table 5:** Scores of FS-14 in the end of treatment

Items	Group A (n = 30)	Group B (n = 30)	*Median (*95%* CI)*	*P-value*
Physical fatigue	5.00 (3.75, 6.00)	6.00 (5.00, 6.25)	− 1.00 (− 2.00, 0.00)	0.003*
Mental fatigue	3.00 (2.00, 3.00)	3.00 (1.75, 4.00)	0.00 (− 1.00, 0.00)	0.542
Total score	8.00 (5.00, 9.00)	9.00 (7.00, 10.00)	− 1.00 (− 3.00, 0.00)	0.012*

Mann-Whitney U test was used for comparing differences between groups to obtain *P-values*. Hodges-Lehmann method was used to calculate median differences and their 95% confidence intervals. Group A: Long-snake-like moxibustion for 60 min; Group B: Long-snake-like moxibustion for 30 min; Data were presented as Median (Q1, Q3); Q: quarter; Q1: 25th percentile; Q3: 75th percentile; 95% CI95% Confidence Interval

* *p* < 0.05

### Secondary outcomes

#### Symptoms scale of spleen-kidney Yang Deficiency

The scores of Symptoms Scale of Spleen-Kidney Yang Deficiency decreased after 4-week treatment (Fig. 4). Compared with Group B, Group A showed significantly lower scores in fear of cold and cold extremities (2.00 vs. 4.00, 95%CI − 2.00 to 0.00, *P* = 0.034), shortness of breath and laziness to speak (2.00 vs. 2.00, 95%CI − 1.00 to 0.00, *P* = 0.045), soreness and weakness of waist and knees (0.00 vs. 2.00, 95%CI − 2.00 to 0.00, *P* = 0.033), lumbar cold pain (1.00 vs. 1.00, 95%CI − 1.00 to 0.00, *P* = 0.028), loose stool (0.00 vs. 1.00, 95%CI − 1.00 to 0.00, *P* = 0.028), cold and more urine volume during night(0.00 vs. 0.00, 95%CI 0.00 to 0.00, *P* = 0.046) and total score (9.80 vs. 13.07, 95%CI − 5.78 to − 0.76, *P* = 0.012) (Table 6).

**Table 6:** Scores of symptoms scale of spleen-kidney Yang deficiency in the end of treatment

Items	Group A (n = 30)	Group B (n = 30)	*Median/MD (*95%CI*)*	*P-value*
Fear of cold and cold extremities	2.00 (1.50, 4.00)	4.00 (2.00, 4.00)	0.00 (− 2.00, 0.00)	0.034*
Lassitude and lack of strength	2.00 (2.00, 2.50)	2.00 (2.00, 2.00)	0.00 (0.00, 0.00)	0.290
Shortness of breath and laziness to speak	2.00 (0.00, 2.00)	2.00 (2.00, 2.00)	0.00 (− 1.00, 0.00)	0.045*
Reduced food intake	0.00 (0.00, 1.25)	0.00 (0.00, 2.00)	0.00 (− 1.00, 0.00)	0.072
Soreness and weakness of waist and knees	0.00 (0.00, 2.00)	2.00 (0.00, 2.00)	0.00 (− 2.00, 0.00)	0.033*
Lumbar cold pain	1.00 (0.00, 1.00)	1.00 (0.00, 2.00)	0.00 (− 1.00, 0.00)	0.028*
Abdominal fullness and distention	1.00 (0.00, 1.00)	1.00 (1.00, 1.00)	0.00 (− 1.00, 0.00)	0.173
Loose stool	0.00 (0.00, 1.00)	1.00 (0.00, 1.00)	0.00 (− 1.00, 0.00)	0.028*
Cold and more urine volume during night	0.00 (0.00, 1.00)	0.00 (0.00, 0.00)	0.00 (0.00, 0.00)	0.046*
Total score	9.80 (4.37)	13.07 (5.29)	− 3.27 (− 5.78, − 0.76)	0.012*

Independent sample T test (for total score) and Mann-Whitney U test (for non-normally distributed items) were used for comparing differences between groups to obtain *P-values*; Hodges-Lehmann method was used to calculate median differences and their 95% confidence intervals. Group A: Long-snake-like moxibustion for 60 min; Group B: Long-snake-like moxibustion for 30 min; Data were presented as mean ± standard deviation (SD) and Median (Q1, Q3); Q: quarter; Q1: 25th percentile; Q3: 75th percentile

*MD* mean difference; *95%CI* 95% Confidence Interval

* *p* < 0.05

### SDS and SAS

SDS and SAS scores of both groups decreased after treatment (Fig. 4), and there were no statistical differences between groups (Table 7).

**Table 7:** Scores of SDS and SAS in the end of treatment

Scales	Group A (n = 30)	Group B (n = 30)	*MD (*95%CI*)*	*P-value*
SDS	43.20 (12.64)	41.87 (11.16)	1.33 (− 4.83, 7.50)	0.667
SAS	39.47 (10.85)	38.00 (9.59)	1.47 (− 3.83, 6.76)	0.581

Independent sample T test was used for comparing differences between groups to obtain *P-values*. Group A: Long-snake-like moxibustion for 60 min; Group B: Long-snake-like moxibustion for 30 min; Data were presented as Mean (SD)

*SD* standard deviation; *MD* mean difference; 95% CI 95% Confidence Interval; *SDS* Self-rating Depression Scale; *SAS* Self-Rating Anxiety Scale

### TTM outcome

When comparing with HCs, statistical differences in ΔT of Groups A and B were obtained at baseline in Middle Jiao (Group A vs. HCs − 0.22 vs. 0.07, *95%CI* 0.10 to 0.64; Group B vs. HCs − 0.33 vs. 0.07, 95%CI 0.15 to 0.79), Lower Jiao (Group A vs. HCs − 0.67 vs. − 0.38, 95%CI 0.06 to 0.76; Group B vs. HCs − 0.81 vs. − 0.38, *95%CI* 0.18 to 0.82), Shenque (CV8) (Group A vs. HCs 0.57 vs. 1.17, 95%CI 0.34 to 1.13; Group B vs. HCs 0.57 vs. 1.17, 95%CI 0.31 to 1.11), neck (Group A vs. HCs 0.68 vs. 1.41, 95%CI 0.29 to 1.46; Group B vs. HCs 0.41 vs. 1.41, 95%CI 0.66 to 1.56), upper arm (Group A vs. HCs − 0.42 vs. 0.24, 95%CI 0.04 to 0.93; Group B vs. HCs − 0.42 vs. 0.24, 95%CI 0.20 to 1.00), thoracic segments (Group A vs. HCs 0.33 vs. 0.76, 95%CI 0.17 to 0.77; Group B vs. HCs 0.31 vs. 0.76, 95%CI 0.31 to 0.93).

After 4-week treatment, statistical differences in ΔT between Group A and HCs were not obtained at the aforementioned sites, while statistical differences in ΔT between Group B and HCs were still obtained in neck (0.85 vs. 1.41, 95%CI 0.09 to 0.88), upper arm (− 0.25 vs. 0.24, 95%CI 0.01 to 0.85) (Table 8).

**Table 8:** Comparison of ΔT between Group A or B and HCs

Sites	Time /ΔT	Group A (n = 30)	Group B (n = 30)	HCs (n = 30)	Median (CI)*	Median (CI)**
Upper Jiao	Baseline	0.32 (− 0.01, 0.56)	0.36 (0.05, 0.54)	0.35 (0.19, 0.58)	0.05 (− 0.18, 0.25)	0.07 (− 0.19, 0.22)
	Week 4	0.43 (0.26, 0.69)	0.43 (0.07, 0.57)	0.35 (0.19, 0.58)	− 0.08 (− 0.25, 0.09)	− 0.02 (− 0.19, 0.21)
Middle Jiao	Baseline	− 0.22 (− 0.71, 0.14)	− 0.33 (− 0.99, 0.09)	0.07 (− 0.11, 0.25)	**0.35 (0.10, 0.64)**	**0.44 (0.15, 0.79)**
	Week 4	0.06 (− 0.02, 0.26)	0.00 (− 0.06, 0.24)	0.07 (− 0.11, 0.25)	0.00 (− 0.18, 0.10)	0.10 (− 0.15, 0.14)
Lower Jiao	Baseline	− 0.67 (− 1.37, − 0.29)	− 0.81 (− 1.89, − 0.41)	− 0.38 (− 0.64, − 0.12)	**0.37 (0.06, 0.76)**	**0.44 (0.18, 0.82)**
	Week 4	− 0.47 (− 0.66, − 0.05)	− 0.57 (− 0.70, − 0.39)	− 0.38 (− 0.64, − 0.12)	0.03 (− 0.21, 0.25)	0.17 (− 0.01, 0.33)
Shenque (CV8)	Baseline	0.57 (− 0.06, 0.91)	0.57 (0.06, 1.17)	1.17 (0.73, 1.78)	**0.74 (0.34, 1.13)**	**0.68 (0.31, 1.11)**
	Week 4	1.29 (0.85, 2.07)	1.12 (0.95, 1.42)	1.17 (0.73, 1.78)	− 0.12 (− 0.54, 0.27)	0.00 (− 0.29, 0.30)
Zhongwan (CV12)	Baseline	− 0.84 (− 1.43, − 0.17)	− 0.91 (− 2.07, − 0.16)	− 0.15 (− 1.19, 0.19)	0.47 (− 0.07, 1.00)	0.65 (0.10, 1.12)
	Week 4	− 0.50 (− 0.92, − 0.11)	− 0.53 (− 0.92, − 0.23)	− 0.15 (− 1.19, 0.19)	0.14 (− 0.47, 0.59)	0.17 (− 0.34, 0.64)
Danzhong (CV17)	Baseline	0.45 (− 0.11, 1.08)	0.35 (− 0.06, 0.80)	0.65 (0.40, 1.11)	0.19 (− 0.16, 0.53)	0.34 (0.05, 0.64)
	Week 4	0.53 (0.10, 0.80)	0.53 (0.08, 0.82)	0.65 (0.40, 1.11)	0.19 (− 0.08. 0.45)	0.26 (− 0.08, 0.54)
Governor vessel	Baseline	0.58 (0.36, 0.91)	0.53 (− 0.01, 0.79)	0.74 (0.40, 1.13)	0.10 (− 0.17, 0.36)	0.31 (0.00, 0.61)
	Week 4	0.60 (0.41, 1.00)	0.68 (0.44, 0.88)	0.74 (0.40, 1.13)	0.08 (− 0.16, 0.32)	0.04 (− 0.17, 0.31)
Pishu (BL20)	Baseline	0.39 (0.09, 0.73)	0.15 (− 0.55, 0.36)	0.40 (0.14, 0.70)	0.02 (− 0.22, 0.30)	0.37 (0.08, 0.74)
	Week 4	0.40 (0.27, 0.74)	0.31 (0.18, 0.42)	0.40 (0.14, 0.70)	− 0.06 (− 0.30, 0.10)	0.08 (− 1.00, 0.30)
Shenshu (BL23)	Baseline	0.45 (0.21, 0.92)	0.16 (− 0.22, 0.54)	0.51 (0.14, 0.97)	0.06 (− 0.24, 0.36)	0.42 (0.13, 0.69)
	Week 4	0.56 (0.51, 0.87)	0.52 (0.25, 0.60)	0.51 (0.14, 0.97)	− 0.11 (− 0.38, 0.16)	0.06 (-0.16, 0.34)
Neck	Baseline	0.68 (− 0.40, 1.48)	0.41 (− 0.08, 0.89)	1.41 (0.74, 1.99)	**0.84 (0.29, 1.46)**	**1.13 (0.66, 1.56)**
	Week 4	0.89 (0.82, 1.79)	0.85 (0.72, 1.31)	1.41 (0.74, 1.99)	0.28 (− 0.17, 0.78)	**0.42 (0.09, 0.88)**
Upper arm	Baseline	− 0.42 (− 1.07, 0.15)	− 0.42 (− 0.81, − 0.18)	0.24 (− 0.42, 0.66)	**0.48 (0.04, 0.93)**	**0.64 (0.20, 1.00)**
	Week 4	− 0.20 (− 0.98, 0.45)	− 0.25 (− 0.98, 0.17)	0.24 (− 0.42, 0.66)	0.34 (− 0.34, 0.82)	**0.50 (0.01, 0.85)**
Thoracic segments	Baseline	0.33 (− 0.24, 0.85)	0.31 (− 0.41, 0.64)	0.76 (0.53, 1.07)	**0.44 (0.17, 0.77)**	**0.60 (0.31, 0.93)**
	Week 4	0.66 (0.54, 1.02)	0.66 (0.51, 0.86)	0.76 (0.53, 1.07)	0.06 (− 0.13, 0.29)	0.11 (− 0.40, 0.33)
Lumbar segments	Baseline	0.35 (− 0.06, 0.82)	0.21 (− 0.12, 0.54)	0.49 (0.22, 0.83)	0.18 (− 0.08, 0.43)	0.35 (0.13, 0.58)
	Week 4	0.63 (0.57, 0.93)	0.59 (0.43, 0.72)	0.49 (0.22, 0.83)	− 0.15 (− 0.37, 0.04)	− 0.09 (− 0.23, 0.11)
Renal region	Baseline	− 0.08 (− 0.77, 0.30)	− 0.32 (− 1.12, 0.03)	0.07 (− 0.13, 0.35)	0.22 (− 0.04, 0.52)	0.49 (0.21, 0.82)
	Week 4	0.11 (− 0.02, 0.41)	0.04 (− 0.20, 0.12)	0.07 (− 0.13, 0.35)	− 0.06 (− 0.26, 0.14)	0.08 (− 0.11, 0.23)

Group A: Long-snake-like moxibustion for 60 min; Group B: Long-snake-like moxibustion for 30 min. Bold data represented statistical differences in ΔT of Groups A and B compared to HCs.  Data were presented as Median (Q1, Q3); Q: quarter; Q1: 25th percentile; Q3: 75th percentile.

*HCs* Healthy control subjects; *95%CI* 95% Confidence Interval

* Group A vs. HCs, ** Group B vs. HCs. Hodges-Lehmann method was used to calculate median differences and their 95% confidence intervals

### Correlation with symptoms and TTM

**Fig. 5:**
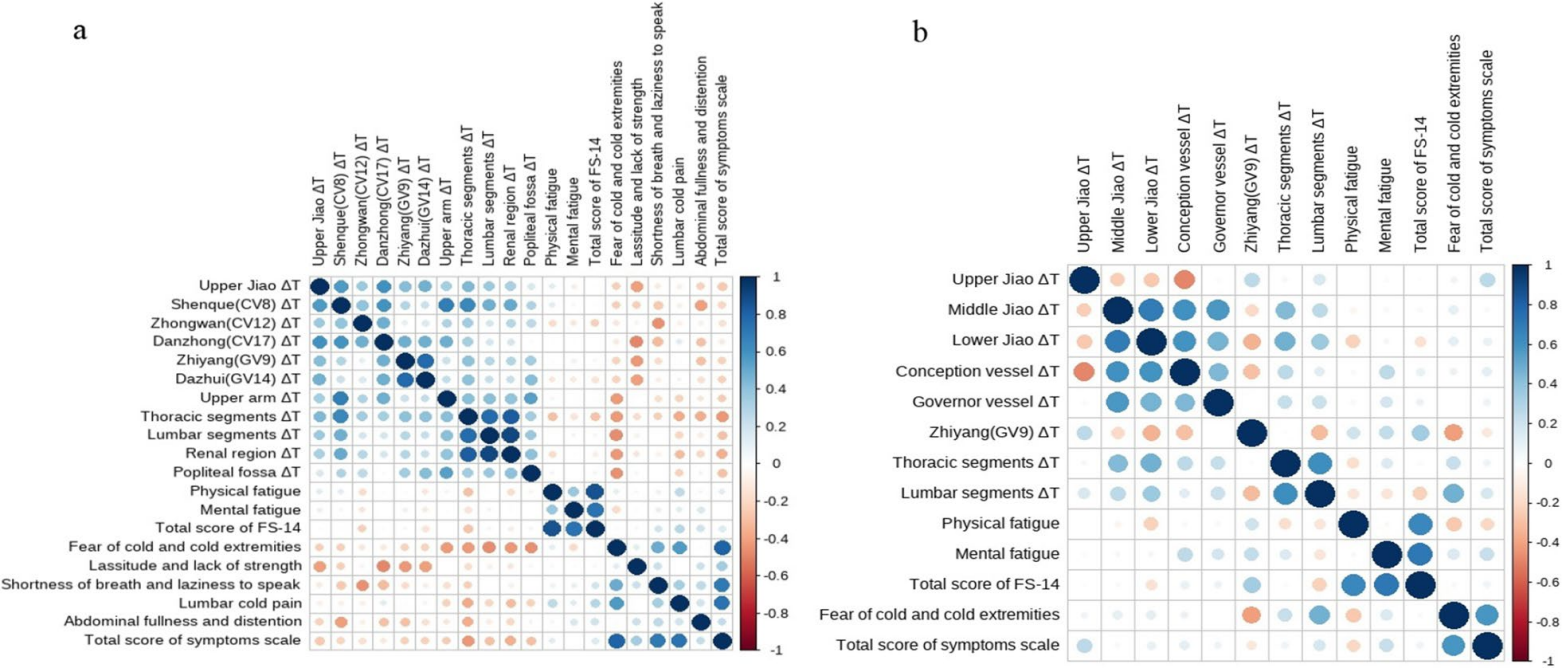
Correlations with score changes and ΔT changes. Score change = week 4-baseline;ΔT change = week 4-baseline; ΔT: temperature difference; **a** Correlations in Group A; **b** Correlations in Group B; Spearman’s correlation was calculated and displayed as corrplot graphs using R package “corrplot”. *r* ranges between − 1 and 1, where − 1 ≤ *r* < 0 means negative correlation, *r* = 0 means no correlation, and 0 < *r* ≤ 1 means positive correlation. Blue circles represent positive correlation and red circles represent negative correlation. The darker the color and the larger the circle, the stronger is the correlation

The two groups had significant correlations between score changes and ΔT changes in TTM (Fig. 5). In Group A, the score change of fear of cold and cold extremities was negatively correlated with ΔT changes of upper arm (*r* = − 0.43, *p* = 0.019), thoracic segments (*r* = − 0.43, *p* = 0.019), lumbar segments (*r* = − 0.46, *p* = 0.010), renal region (*r* = − 0.44, *p* = 0.016), popliteal fossa (*r* = − 0.46, *p* = 0.011); the score change of lassitude and lack of strength was negatively correlated with ΔT changes of Upper Jiao (*r* = − 0.42, *p* = 0.022), Danzhong (CV17) (*r* = − 0.49, *p* = 0.006), Zhiyang (GV9) ( *r* = − 0.44, *p* = 0.016), Dazhui (GV14) (*r* = − 0.42, *p* = 0.023); the score change of shortness of breath and laziness to speak was negatively correlated with ΔT change of Zhongwan (CV12) (*r* = − 0.46, *p* = 0.011); the score change of lumbar cold pain was negatively correlated with ΔT change of thoracic segments (*r* = − 0.38, *p* = 0.041); the score change of abdominal fullness and distention was negatively correlated with ΔT changes of Shenque (CV8) (*r* = − 0.42, *p* = 0.022), thoracic segments (*r* = − 0.37, *p* = 0.047); the total score change of Spleen-Kidney Yang Deficiency Symptoms Scale was negatively correlated with ΔT change of thoracic segments (*r* = − 0.44, *p* = 0.016). In Group B, only the score change of fear of cold and cold extremities was negatively correlated with ΔT change of Zhiyang (GV9) (*r* = − 0.41, *p* = 0.024).

### Sensitivity analysis

Sensitivity analysis performed by the per protocol set showed similar results as the intention-to-treat analysis (Additional file 1).

## Discussion

Due to the non-organic pathophysiology, CFS is associated with suboptimal health status [29]. The present treatment of CFS is given based on limited or even contradictory evidence from clinical studies [30–32]. CFS is named as fatigue syndrome in TCM theory and its main reason is believed to be Yang-qi deficiency. Especially Spleen and Kidney Yang are thought to be the vital motivity, and organs will become hypofunctional once Yang qi decreases. Therefore, Spleen-Kidney Yang Deficiency Syndrome is a commonly used visceral pattern in clinical diagnosis of CFS [33, 34]. Moxibustion therapy has been applied in Yang deficiency patients for thousands of years in China and it is used widely to relieve fatigue symptoms [35]. A systematic review has suggested that moxibustion has more advantages than acupuncture and drugs in relieving fatigue symptoms and improving clinical efficacy [36]. As a unique traditional moxibustion therapy, Long-snake-like moxibustion has shown to be a safe and effective therapy for relieving fatigue and improving Yang deficiency constitution [14, 37].

The dose–effect relationship is a crucial issue in the moxibustion research, with key factors such as treatment duration, frequency, extent, and amount of moxa cones/sticks determining the moxibustion dose. However, the relationship between the dose of moxibustion stimulation and the resulting therapeutic efficacy has not yet been established. Previous studies have shown that the above dose factors affect efficacy to varying degrees in different diseases. For instance, in the study of treating diarrhea-predominant irritable bowel syndrome, aconite cake-separated moxibustion with a treatment regimen of 3 sessions per week and 1 cone per session appeared to produce better therapeutic effects compared to a regimen of 6 sessions per week and 2 cones per session [18]. Another study on suspended moxibustion for focal cerebral ischemia/reperfusion injury found that 35-min moxibustion indicated a greater anti-apoptotic effect than 15-min moxibustion [17]. In the treatment of depression-like behavior disorder in rats, prolonged duration of moxibustion under optimal extent could increase the level of 5-HT [16]. Furthermore, in the treatment of chronic neck pain using direct moxibustion with small moxa cones, there was a trend of improved effectiveness with an increase in the amount of moxa cones when the other dose factors were constant [15].

The duration of Long-snake-like moxibustion is a key factor in optimizing efficacy, but the treatment duration for CFS varies [38, 39]. In our 4-week trial with 3 treatments per week, we compared the dose–effect relationship of the therapy using treatment durations of 30 and 60 min. Our findings suggest that 60-min Long-snake-like moxibustion is more effective in relieving fatigue and improving symptoms of Spleen-Kidney Yang Deficiency compared to 30-min Long-snake-like moxibustion. Specifically, the fatigue was relieved primarily by improving physical fatigue rather than improving mental fatigue, and the TCM pattern was improved primarily by alleviating main symptoms (such as fear of cold and cold extremities, soreness and weakness of waist and knees, lumbar cold pain, etc.), suggesting that 60-min Long-snake-like moxibustion therapy has a better ability to tonify Yang qi and improve the fatigue state. However, there was no statistically significant between-group difference in lassitude and lack of strength in the Symptoms Scale of Spleen-Kidney Yang Deficiency, which was different from the result obtained with FS-14. This discrepancy may be due to the fact that fatigue is a multidimensional and subjective sense of physical, mental, or emotional tiredness [40]. Lassitude and lack of strength is only a component item in the Symptoms Scale of Spleen-Kidney Yang Deficiency and it is relatively simple and unidimensional. In contrast, FS-14 is an internationally recognized measurement tool for evaluating fatigue symptoms in clinics, providing a more comprehensive reflection of fatigue severity [22, 41]. It could measure fatigue across multiple dimensions with better reliability and validity, offering a clearer understanding of the type of fatigue experienced and improved assessment precision compared to single-item measures [42]. Besides, statistically significant between-group differences in SDS and SAS were not observed, which may be associated with the relatively short course of treatment and no follow-up period. The moxibustion effects on mental fatigue and mental distress require further study.

A large body of evidence has showed that diseases or deviation from normal functioning are accompanied by temperature changes in the body, which, in turn, affect the temperature of the skin. Infrared imaging (IR) has proven to be a useful method for diagnosing signs of certain diseases by measuring the local skin temperature. As a new medical IR technology, TTM enables contactless and non-invasive identification of abnormal heat sources within the human body by analyzing thermographic data and surface temperature distribution [43]. Since its invention, TTM has been applied in disease diagnosis such as oncology (breast, skin, etc.), vascular disorders (diabetes, deep venous thrombosis), and monitoring the efficacy of therapeutic drugs, etc. [44–48]. Therefore, we combined measurements of the subjective patient-reported scales with objective TTM technology in this trail. Unlike previous study that only used objective technique to quantify muscle fatigue [49], our trial took a comprehensive approach. We not only employed subjective scales to evaluate fatigue levels and Spleen-Kidney Yang Deficiency symptoms before and after treatment but also utilized objective technique (TTM) to assess thermal energy metabolism changes in CFS patients and compared them with those of HCs. The aim of this trial was to quantify the therapeutic effect of Long-snake-like moxibustion, evaluate the thermal energy metabolism under different physiological/pathological conditions and different durations of moxibustion, and obtain the optimal dose–effect of Long-snake-like moxibustion.

According to the results, most thermal radiation values in CFS patients were obviously lower than those in HCs at baseline, and the ΔTs in CFS patients were statistically different from those of HCs in Middle Jiao, Lower Jiao, Shenque (CV8), neck, upper arm, thoracic segments. Some studies have shown that irregularities in energy metabolism [50], impaired cell metabolism [51], activated immune system [52], viral infection [53], impaired functioning in the hypothalamic-pituitary-adrenal axis and neuroendocrine dysregulation [54] have been considered possible causes of CFS. An animal experiment indicated that fatigued rats would have a deterioration in energy metabolism [55]. Since TTM images present the level of energy metabolism by displaying the thermal radiation value distribution [56], we found that most CFS patients were in a state of low energy metabolism. After 4-week Long-snake-like moxibustion, thermal radiation values increased at both directly and indirectly stimulated sites by moxibustion, indicating that Long-snake-like moxibustion could transfer and penetrate heat to far and deep sites, increase metabolic heat in different sites and regions of the body, and improve the low energy metabolism state. This heat energy transfer may be the underlying mechanism of moxibustion therapy [57]. Moreover, at the end of 4-week treatment, there were no statistically significant differences in ΔTs between the 60-min Long-snake-like moxibustion group and HCs in the above sites, whereas the ΔTs in neck and upper arm still showed statistically significant differences between the 30-min Long-snake-like moxibustion group and HCs. This suggests that 60-min Long-snake-like moxibustion has a more pronounced ability to increase thermal radiation values and demonstrates a closer trend to that of HCs. The TTM results were consistent with the subjective scales results, suggesting that 60-min Long-snake-like moxibustion had a more significant effect than 30-min moxibustion.

It is worth mentioning that ΔT changes were negatively correlated with the score changes, indicating that the more thermal radiation values increased, the better the CFS symptoms improved. This suggests that the increase in thermal radiation values may be an indirect reflection of warming and tonifying Yang. ΔT changes in Upper Jiao, Shenque (CV8), Zhongwan (CV12), Danzhong (CV17), Zhiyang (GV9), Dazhui (GV14), upper arm, thoracic segments, lumbar segments, renal region, popliteal fossa strongly correlated with the improvement of Spleen-Kidney Yang Deficiency symptoms. This suggests that Zhiyang (GV19), Dazhui (GV14), thoracic segments, lumbar segments, renal region on the Governor vessel are key acupoints and regions with the Yang-tonifying effect. Other randomized controlled trials have also shown that moxibustion on the Governor vessel has good therapeutic effects on Yang deficiency diseases [13, 58]. The improvement of Yang deficiency symptoms is also related to the fact that Long-snake-like moxibustion stimulates Yang of the whole body to a greater extent by transferring heat to the extremities and penetrating heat to Upper Jiao and acupoints on the Conception vessel. This heat transfer and penetration of moxibustion are considered manifestations of Deqi [59, 60], which is believed to be an important parameter in acupuncture and moxibustion treatment [61]. The number of correlations was significantly greater in the 60-min Long-snake-like moxibustion group than in the 30-min Long-snake-like moxibustion group. Specifically, there were correlations between Upper Jiao, Shenque (CV8), Zhongwan (CV12), Danzhong (CV17), Zhiyang (GV9), Dazhui (GV14), upper arm, thoracic segments, lumbar segments, renal region, popliteal fossa and fear of cold and cold extremities, lassitude and lack of strength, shortness of breath and laziness to speak, lumbar cold pain, abdominal fullness and distention, symptoms total score in the 60-min Long-snake-like moxibustion group. However, there was only one correlation between Zhiyang (GV9) and fear of cold and cold extremities in the 30-min Long-snake-like moxibustion group. This result suggests that the longer the duration of moxibustion, the greater the dose of heat stimulation. As the heat transfers further and penetrates deeper, more acupoints and regions are mobilized to tonify Yang. In the same course of treatment, 60-min duration generates a greater dose of moxibustion and a stronger effect on tonifying Yang.

One of the limitations of our trial is the small sample size. Considering the pathogenesis characteristic of CFS [2], only female subjects were recruited and examined. Further trials could enlarge the sample size or recruit male subjects to reduce the selection bias. Another limitation is that there were only two time points for TTM scanning in this trial. Increasing the course of treatment and adding additional scanning time points in further trials may be beneficial for analyzing the dynamic changes of dose–effect of Long-snake-like moxibustion and helping deeply explore the saturated dose rule of Long-snake-like moxibustion.

## Conclusions

This trial evaluated the effect difference of Long-snake-like moxibustion with different dose of 60-m and 30-m duration, measured by subjective traditional scales and objective medical infrared imaging technology. In the same treatment course, 60-min Long-snake-like moxibustion is superior to 30-min Long-snake-like moxibustion in relieving physical fatigue and overall fatigue status, improving Spleen-Kidney Yang Deficiency symptoms, and raising the low heat state to the level of healthy people. This trial provides evidence for selecting the appropriate treatment duration for Long-snake-like moxibustion in treating CFS, and enriches the content of dose–effect research on moxibustion therapy.

## Supplementary Information

**Additional file 1:** Sensitivity analysis.

## Data Availability

The datasets used and/or analyzed during the current study are available from the corresponding author on reasonable request.

## Abbreviations

CFS: Chronic fatigue syndrome
TCM: Traditional Chinese medicine
GV: Governor Vessel
TTM: Thermal texture maps
HCs: Healthy control subject
FS-14: Fatigue scale-14
SDS: Self-rating depression scale
SAS: Self-rating anxiety scale
SD: Standard deviation
Q1: 25th percentile
Q3: 75th percentile
CI: Certificate interval
CRF: Case report form
ITT: Intention-to-treat
BMI: Body mass index
CV: Conception vessel
T: Thermal radiation value
