# Supplementary material for: Dose–effect of long-snake-like moxibustion for chronic fatigue syndrome: a randomized controlled trial
Source: J Transl Med. 2023 Jul 3;21:430. doi: 10.1186/s12967-023-04250-z (PMC10316615; doi:10.1186/s12967-023-04250-z)
Supplement: Supplementary file 1 — Additional file 1: Sensitivity analysis. [file 12967_2023_4250_MOESM1_ESM.docx]

**Additional file 1** Sensitivity analysis

(1) Scores of FS-14 in the end of treatment (per protocol set analysis)

| Items | Group A (*n* = 28) | Group B (*n* = 29) | *Median (95% CI)* | *P-value* |
| --- | --- | --- | --- | --- |
| Physical fatigue | 5.00 (3.75, 6.00) | 6.00 (5.00, 6.25) | -1.00 (-2.00, 0.00) | 0.007* |
| Mental fatigue | 3.00 (2.00, 3.00) | 3.00 (1.75, 4.00) | 0.00 (-1.00, 0.00) | 0.519 |
| Total score | 8.00 (5.00, 9.00) | 9.00 (7.00, 10.00) | -1.00 (-3.00, 0.00) | 0.016* |

(2) Scores of Symptoms Scale of Spleen-kidney Yang Deficiency in the end of treatment (per protocol set analysis)

| Items | Group A (n = 28) | Group B (n = 29) | Median/MD(95%CI) | P-value |
| --- | --- | --- | --- | --- |
| Fear of cold and cold extremities | 2.00 (1.00, 4.00) | 4.00 (2.00, 4.00) | -2.00 (-2.00, 0.00) | 0.025* |
| Lassitude and lack of strength | 2.00 (2.00, 3.00) | 2.00 (2.00, 2.00) | 0.00 (0.00, 0.00) | 0.302 |
| Shortness of breath and laziness to speak | 2.00 (0.00, 2.00) | 2.00 (2.00, 2.00) | 0.00 (-1.00, 0.00) | 0.077 |
| Reduced food [intake](https://dict.bioon.com/detail.asp?id=278b680394) | 0.00 (0.00, 0.50) | 0.00 (0.00, 2.00) | 0.00 (0.00, 0.00) | 0.110 |
| Soreness and weakness of waist and knees | 0.00 (0.00, 2.00) | 2.00 (0.00, 2.00) | 0.00 (-2.00, 0.00) | 0.067 |
| Lumbar cold pain | 1.00 (0.00, 1.00) | 1.00 (0.00, 1.75) | 0.00 (-1.00, 0.00) | 0.057 |
| Abdominal fullness and distention | 1.00 (0.00, 1.00) | 1.00 (1.00, 1.00) | 0.00 (-1.00, 0.00) | 0.316 |
| Loose stool | 0.00 (0.00, 1.00) | 1.00 (0.25, 1.00) | 0.00 (-1.00, 0.00) | 0.029* |
| Cold and more urine volume during night | 0.00 (0.00, 1.00) | 0.00 (0.00, 0.00) | 0.00 (0.00, 0.00) | 0.054 |
| Total score | 9.83 (4.45) | 13.18 (5.45) | -3.35 (-5.99, -0.71) | 0.014* |

(3) Scores of SDS and SAS in the end of treatment (per protocol set analysis)

| Scales | Group A (*n* = 28) | Group B (*n* = 29) | *MD(95%CI)* | *P-value* |
| --- | --- | --- | --- | --- |
| SDS | 43.48 (12.80) | 40.86 (10.69) | 2.63 (-3.64, 8.89) | 0.404 |
| SAS | 39.76 (10.92) | 37.36 (9.60) | 2.40 (-3.07, 7.87) | 0.382 |

(4) Comparison of ΔT between Group A or B and HCs (per protocol set analysis)

| Sites | Time /ΔT | Group A  (*n* = 28) | Group B  (*n* = 29) | HCs  (*n* = 30) | *Median (CI)** | *Median (CI)*** |
| --- | --- | --- | --- | --- | --- | --- |
| Upper Jiao | Baseline | 0.32 (-0.01, 0.56) | 0.36 (0.05, 0.54) | 0.35 (0.19, 0.58) | 0.05 (-0.18, 0.25) | 0.07 (-0.19, 0.22) |
|  | Week 4 | 0.44 (0.25, 0.71) | 0.44 (0.29, 0.60) | 0.35 (0.19, 0.58) | -0.08 (-0.26, 0.09) | -0.07 (-0.20, 0.15) |
| **Middle Jiao** | Baseline | -0.22 (-0.71, 0.14) | -0.33 (-0.99, 0.09) | 0.07 (-0.11, 0.25) | **0.35 (0.10, 0.64)** | **0.44 (0.15, 0.79)** |
|  | Week 4 | 0.06 (-0.04, 0.29) | 0.00 (-0.07, 0.21) | 0.07 (-0.11, 0.25) | 0.00 (-0.18, 0.12) | 0.04 (-0.11, 0.15) |
| **Lower Jiao** | Baseline | -0.67 (-1.37, -0.29) | -0.81 (-1.89, -0.41) | -0.38 (-0.64, -0.12) | **0.37 (0.06, 0.76)** | **0.44 (0.18, 0.82)** |
|  | Week 4 | -0.47 (-0.63, -0.04) | -0.52 (-0.61, -0.38) | -0.38 (-0.64, -0.12) | 0.01 (-0.22, 0.24) | 0.15 (-0.03, 0.33) |
| **Shenque**  **(CV8)** | Baseline | 0.57 (-0.06, 0.91) | 0.57 (0.06, 1.17) | 1.17 (0.73, 1.78) | **0.74 (0.34, 1.13)** | **0.68 (0.31, 1.11)** |
|  | Week 4 | 1.22 (0.84, 2.02) | 1.12 (0.92, 1.40) | 1.17 (0.73, 1.78) | -0.08 (-0.49, 0.27) | 0.10 (-0.26, 0.34) |
| Zhongwan  (CV12) | Baseline | -0.84 (-1.43, -0.17) | -0.91 (-2.07, -0.16) | -0.15 (-1.19, 0.19) | 0.47 (-0.07, 1.00) | 0.65 (0.10, 1.12) |
|  | Week 4 | -0.46 (-0.92, -0.03) | -0.62 (-0.92, -0.39) | -0.15 (-1.19, 0.19) | 0.13 (-0.53, 0.58) | 0.29 (-0.23, 0.67) |
| Danzhong  (CV17) | Baseline | 0.45 (-0.11, 1.08) | 0.35 (-0.06, 0.80) | 0.65 (0.40, 1.11) | 0.19 (-0.16, 0.53) | 0.34 (0.05, 0.64) |
|  | Week 4 | 0.53 (0.10, 0.80) | 0.44 (0.03, 0.79) | 0.65 (0.40, 1.11) | 0.18 (-0.11. 0.45) | 0.31 (-0.10, 0.59) |
| Governor vessel | Baseline | 0.58 (0.36, 0.91) | 0.53 (-0.01, 0.79) | 0.74 (0.40, 1.13) | 0.10 (-0.17, 0.36) | 0.31 (0.00, 0.61) |
|  | Week 4 | 0.68 (0.43, 1.01) | 0.68 (0.44, 0.86) | 0.74 (0.40, 1.13) | 0.05 (-0.19, 0.31) | 0.04 (-0.15, 0.29) |
| Pishu  (BL20) | Baseline | 0.39 (0.09, 0.73) | 0.15 (-0.55, 0.36) | 0.40 (0.14, 0.70) | 0.08 (-0.31, 0.10) | 0.37 (0.08, 0.74) |
|  | Week 4 | 0.40 (0.30, 0.75) | 0.31 (0.18, 0.40) | 0.40 (0.14, 0.70) | -0.06 (-0.30, 0.10) | 0.11 (-0.50, 0.32) |
| Shenshu  (BL23) | Baseline | 0.45 (0.21, 0.92) | 0.16 (-0.22, 0.54) | 0.51 (0.14, 0.97) | -0.13 (-0.40, 0.12) | 0.42 (0.13, 0.69) |
|  | Week 4 | 0.56 (0.52, 0.87) | 0.52 (0.25, 0.56) | 0.51 (0.14, 0.97) | -0.11 (-0.38, 0.16) | 0.08 (-0.14, 0.36) |
| **Neck** | Baseline | 0.68 (-0.40, 1.48) | 0.41 (-0.08, 0.89) | 1.41 (0.74, 1.99) | **0.84 (0.29, 1.46)** | **1.13 (0.66, 1.56)** |
|  | Week 4 | 0.89 (0.85, 1.81) | 0.85 (0.71, 1.19) | 1.41 (0.74, 1.99) | 0.25 (-0.22, 0.73) | **0.47 (0.12, 0.94)** |
| **Upper arm** | Baseline | -0.42 (-1.07, 0.15) | -0.42 (-0.81, -0.18) | 0.24 (-0.42, 0.66) | **0.48 (0.04, 0.93)** | **0.64 (0.20, 1.00)** |
|  | Week 4 | -0.20 (-0.98, 0.57) | -0.25 (-0.98, 0.11) | 0.24 (-0.42, 0.66) | 0.26 (-0.40, 0.78) | **0.50 (0.04, 0.84)** |
| **Thoracic segments** | Baseline | 0.33 (-0.24, 0.85) | 0.31 (-0.41, 0.64) | 0.76 (0.53, 1.07) | **0.44 (0.17, 0.77)** | **0.60 (0.31, 0.93)** |
|  | Week 4 | 0.66 (0.53, 1.06) | 0.61 (0.50, 0.77) | 0.76 (0.53, 1.07) | 0.06 (-0.14, 0.29) | 0.15 (-0.01, 0.41) |
| Lumbar segments | Baseline | 0.35 (-0.06, 0.82) | 0.21 (-0.12, 0.54) | 0.49 (0.22, 0.83) | 0.18 (-0.08, 0.43) | 0.35 (0.13, 0.58) |
|  | Week 4 | 0.63 (0.58, 0.94) | 0.58 (0.43, 0.64) | 0.49 (0.22, 0.83) | -0.17 (-0.39, 0.04) | -0.06 (-0.22, 0.12) |
| Renal region | Baseline | -0.08 (-0.77, 0.30) | -0.32 (-1.12, 0.03) | 0.07 (-0.13, 0.35) | 0.22 (-0.04, 0.52) | 0.49 (0.21, 0.82) |
|  | Week 4 | 0.11 (-0.01, 0.42) | 0.04 (-0.18, 0.12) | 0.07 (-0.13, 0.35) | -0.08 (-0.27, 0.12) | 0.08 (-0.10, 0.23) |

(5) Correlation figures with symptoms and TTM (per protocol set analysis)


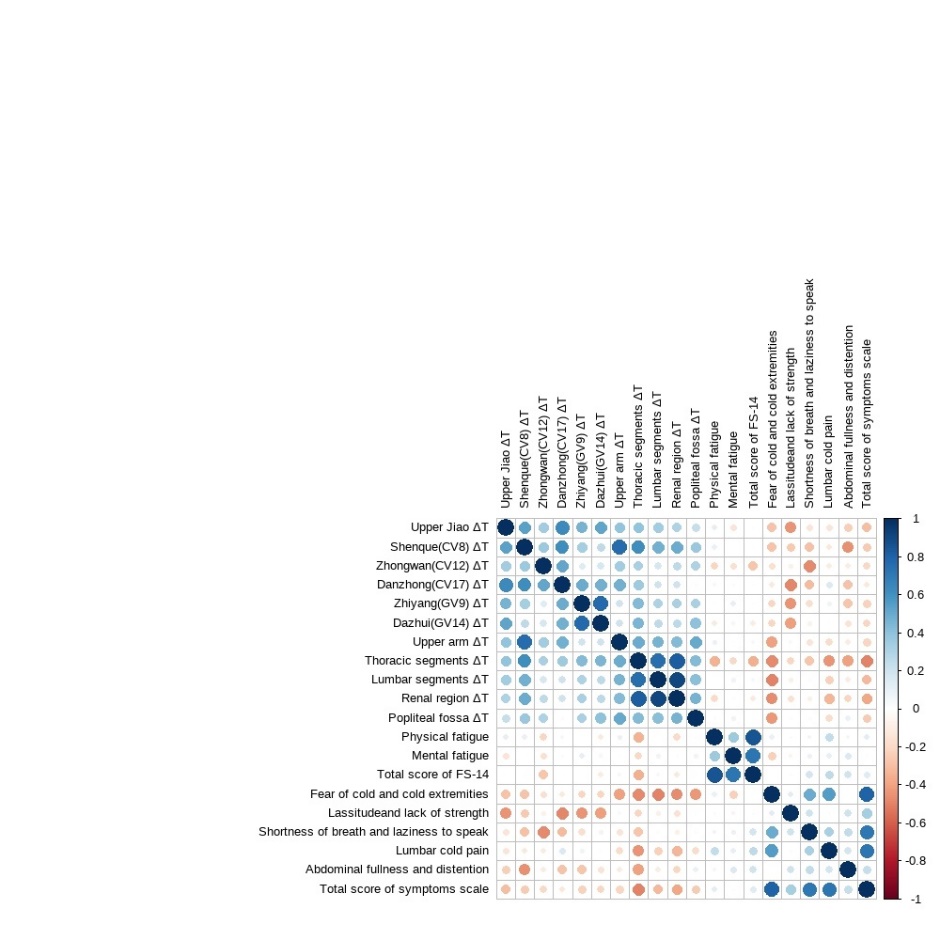

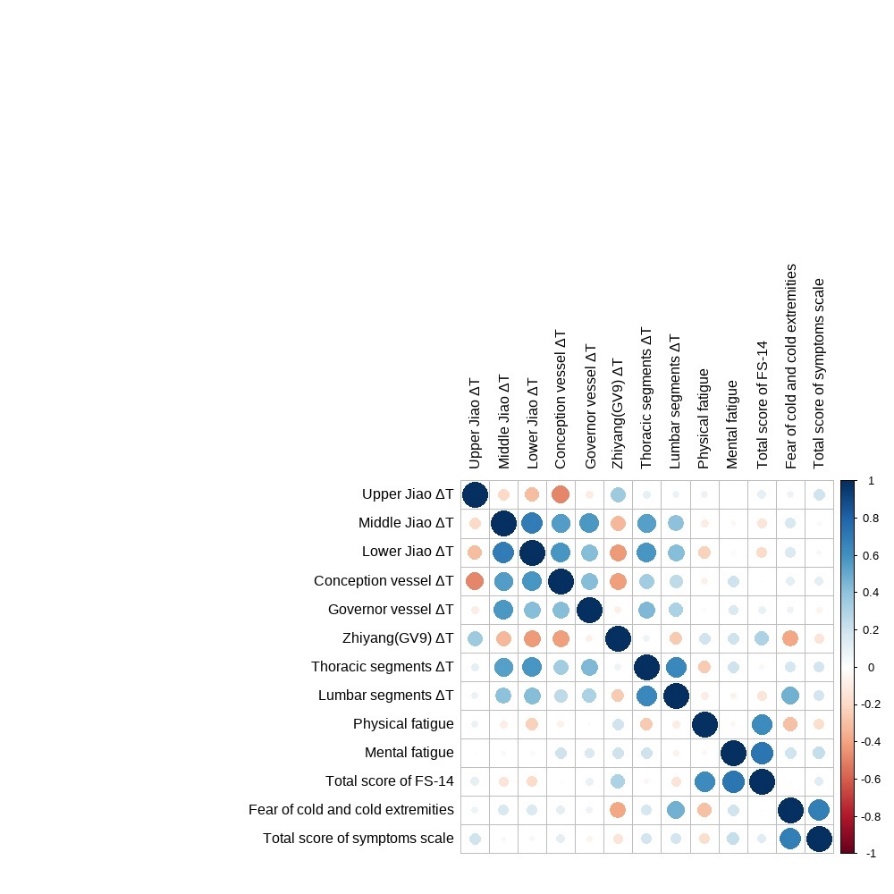


A

B
